# Health hazards of hexavalent chromium (Cr (VI)) and its microbial reduction

**DOI:** 10.1080/21655979.2022.2037273

**Published:** 2022-02-14

**Authors:** Pooja Sharma, Surendra Pratap Singh, Sheetal Kishor Parakh, Yen Wah Tong

**Affiliations:** a Environmental Research Institute, National University of Singapore, Singapore; b Energy and Environmental Sustainability for Megacities (E2S2) Phase II, Campus for Research Excellence and Technological Enterprise (Create), Singapore; c Plant Molecular Biology Laboratory, Department of Botany, Dayanand Anglo-Vedic (PG) College, Chhatrapati Shahu Ji Maharaj University, Kanpur India; d Department of Chemical and Biomolecular Engineering, National University of Singapore, Singapore

**Keywords:** Microbial bioremediation, biosorption, environmental contaminates, health hazards, chromium toxicity

## Abstract

Industrial effluents/wastewater are the main sources of hexavalent chromium (Cr (VI)) pollutants in the environment. Cr (VI) pollution has become one of the world’s most serious environmental concerns due to its long persistence in the environment and highly deadly nature in living organisms. To its widespread use in industries Cr (VI) is highly toxic and one of the most common environmental contaminants. Cr (VI) is frequently non-biodegradable in nature, which means it stays in the environment for a long time, pollutes the soil and water, and poses substantial health risks to humans and wildlife. In living things, the hexavalent form of Cr is carcinogenic, genotoxic, and mutagenic. Physico-chemical techniques currently used for Cr (VI) removal are not environmentally friendly and use a large number of chemicals. Microbes have many natural or acquired mechanisms to combat chromium toxicity, such as biosorption, reduction, subsequent efflux, or bioaccumulation. This review focuses on microbial responses to chromium toxicity and the potential for their use in environmental remediation. Moreover, the research problem and prospects for the future are discussed in order to fill these gaps and overcome the problem associated with bacterial bioremediation’s real-time applicability.

## Graphical abstract

**Figure:**
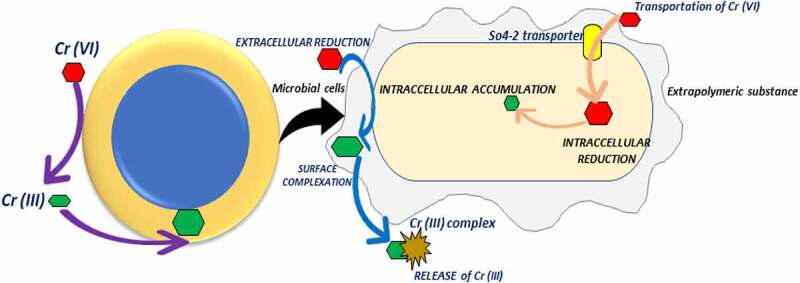

## 1. Introduction

Chromium (Cr) is a naturally occurring element present in volcanic dust, rocks, and soil [1]. Cr has high redox potential, and it can exist in various oxidation states ranging from (-II) to (IV). However, its most stable forms are trivalent Cr (Cr (III)) and hexavalent Cr (Cr (VI)) [2]. The physical, chemical, and toxicological properties of Cr (III) and Cr (IV) vary considerably. While Cr (III) is usually found in nature in the form of ore such as ferrochromite, Cr (VI) is mostly generated from anthropogenic activities and is highly toxic to living organisms [3]. Cr (III) exists as an insoluble hydroxide cation whereas Cr (VI) is an oxyanion species occurring in the form of divalent chromate, dichromate, or monovalent chromate depending on the solution pH [4]. Cr (VI) is the most mobile form of Cr in the aquatic environment due to its relatively higher water solubility. Chromium is a versatile element and has been used in many industrial applications since its discovery in 1797. Chromium compounds are often used in chrome plating, dye manufacturing, textile industry, aircraft industry, leather tanning, wood preservation, and mud drilling. Chromates, dichromates, chromic acid, chromic sulfate and, chromic oxides are examples of industrially relevant chromium compounds. These chromium compounds are generally produced from the mining and treatment of chromite ore. However, such mining and industrial activities generate considerable volumes of solid and liquid chromium-rich waste, as well as result in its atmospheric emissions [5]. In addition to mining and industrial activities, natural rocks such as ultramafic and mafic rocks are also a geogenic source of Cr (VI) in groundwater [4–6]. Increased concentrations of Cr (VI) have been observed in water reservoirs linked with ultramafic aquifers in California, Mexico, Brazil, Italy, Argentina, and Greece [7–10]. Moreover, Cr (VI) concentrations in igneous and meta-volcanic groundwater, as well as aquifers associated with mixed or more felsic igneous and metamorphic formations, are relatively high in North Carolina (up to 25 g/L). Environmental contamination by Cr (VI) has become a worldwide concern. In many places of the world, industries have disposed of hazardous waste in ways that benefit their bottom lines, such as illegal dumpsites, at the expense of the environment and human health. These dumpsites are the primary source of Cr pollution and long-term damage to groundwater.

The following are some of the most important sources of Cr (VI)

- Dyes, paints, inks, and polymers containing chromate pigments.
- Chrome plating is the process of putting chromium metal onto an item’s surface using a chromic acid solution.
- Particles produced while ferrochromium ore smelting.
- Welding fumes from stainless steel and nonferrous chromium alloys

Cr (VI) is classified as a group 1 carcinogen by the World Health Organization (WHO). The maximum allowed concentration of chromium in drinking water is set at 50 ug/L by the drinking water guideline. In the United States and Canada, average Cr (VI) levels in drinking water range from 0.2 to 2 µg-Cr (VI)/L [11,12]. Although the US Environmental Protection Agency (US EPA) acknowledges Cr (VI) as a harmful element. Only total chromium (Cr(T) is included in the drinking water standard, with a maximum pollutant level of 100 µg/L. To avoid the ill effects of Cr (VI) on human health, there is an urgent need to implement strict environmental restrictions to limit the amount of Cr (VI) that can be released into the environment. Several treatment procedures for Cr (VI) removal from wastewater exist, including adsorption, chemical precipitation, ion exchange, electrocoagulation, membrane separation, and electrodialysis [13]. Amongst which, chemical precipitation is the most common approach to Cr (VI) elimination. Calcium hydroxide, magnesium oxide, sodium hydroxide, and calcium magnesium carbonate are examples of some chemical precipitators used in Cr (VI) removal. All criteria or elements that affect precipitation include the type of precipitation agent, sludge volume, speed of agitation, pH, mixing duration, and complexing agents [14,15]. Advanced treatment procedures including reverse osmosis, ion exchange, membrane filtering, electrocoagulation, and electrodialysis are successful at eliminating Cr (VI), but they are costly and produce concentrated wastes that must be treated and disposed of later [16]. Bioremediation is emerging as may be an effective way technique to remove Cr (VI) from industrial effluents. Chromium bioremoval has been documented using a variety of fungal and bacterial species. *Streptomyces rimosus, Actinomycetes*, and *Streptomyces griseus* both showed promise in removing Cr (VI) from industrial effluent [17]. However, chitosan, rice husk, waste tea leaves, pomegranate husk, neem leaves, coconut shell, orange peel, watermelon rind, sawdust, and banana rachis are low-cost farm wastes with adsorption abilities to remove Cr (VI) from wastewater [18–23]. There is not a thorough investigation on the use of chromium-resistant bacteria to remediate chrome-polluted effluent. Hexavalent chromium is a well-known environmental contaminant that has the potential to cause cancer, teratogenicity, and mutation [24]. The goal of this review is to reveal the harmful effects of Cr (VI), as well as ways for remediating polluted sites by using microbes to absorb and break down Cr (VI) pollutants.

## 2. Hexavalent chromium (Cr (VI)) effect on human health

Heavy metal contamination has become a severe environmental hazard worldwide in recent decades [25,26]. Hexavalent chromium [Cr (VI)] is a global environmental pollutant that increases the risk for several types of cancers and is increasingly being recognized as a neurotoxicant [24]. Several kinds of plants and microbes play crucial roles in the removal of toxic metals from contaminated sites [27–32]. Cr (VI) and its metabolites, particularly chromates, take a distinct route into the human body. The main routes of Cr (VI) exposure include inhalation, ingestion, and skin contact. Depending on the duration, Cr (VI) exposure can be classified as acute (14 days), intermediate (75–364 days), and chronic (365 days) [33,34]. Cr (VI) causes toxicity in a variety of ways. It can reduce immune system activity or efficiency, compete with enzyme activity cofactor fixation sites, suppress important enzymes such as oxidative phosphorylation, and cause changes in cell architecture, notably in the lipoprotein region of the membrane. Nasal irritation and ulceration, hypersensitivity reactions and contact dermatitis, acute bronchitis and emphysema, liver and kidney disease, lung and skin cancer, internal bleeding, and DNA damage are all caused by the interaction of Cr (VI) with the DNA-polymerase enzyme [35]. Cr (VI) rapidly enters cells, but it needs to pass through several stages in the bloodstream before becoming Cr (III) in the internal organs. The Cr (VI) ion is excreted from the body, whereas the chromate ion is carried to the cell via a transport pathway that also involves the ions sulfate and phosphate. Such ions can induce oxidative stress in cells, which has been associated with a variety of chronic, cardiovascular, neurodegenerative diseases. Cr (VI) damages cells in a variety of ways, such as increased oxidative stress, the creation of DNA adducts, and chromosome breakups [36]. The World Health Organization’s International Agency for Data on Cancer (IARC) has classified Cr (VI) compounds as group one human carcinogens with several complex modes of action based on epidemiological research tying Cr (VI) to lung cancer [37,38]. Eardrum perforation, irritations, allergies, eczema, respiratory tract issues, skin irritations, ulceration, and lung cancer have all been linked to human exposure to Cr (VI) [39]. At various phases, Cr (VI) radiation can produce cytotoxic, mutagenic, and DNA mutations, as well as carcinogenic effects of Cr (VI)-containing compounds, chromosomal damage, and oxidative protein changes [40]. Nasal lining nose ulcers, irritation, anemia, and ulcers in the small intestine and stomach, and other respiratory problems like nasal blockage, coughing, wheezing, and face erythema, can all be caused by inhaling a high amount of hexavalent chromium.

Hexavalent chromium exposure at work may result in the following health consequences:

- If hexavalent chromium is inhaled in high quantities, it can cause irritation or injury to the nostrils, throat, and lungs (respiratory tract).
- Lung cancer in workers exposed to hexavalent chromium in the air.
- If hexavalent chromium comes into touch with organs in high amounts, it might cause irritation or injury.

**Figure 1.**
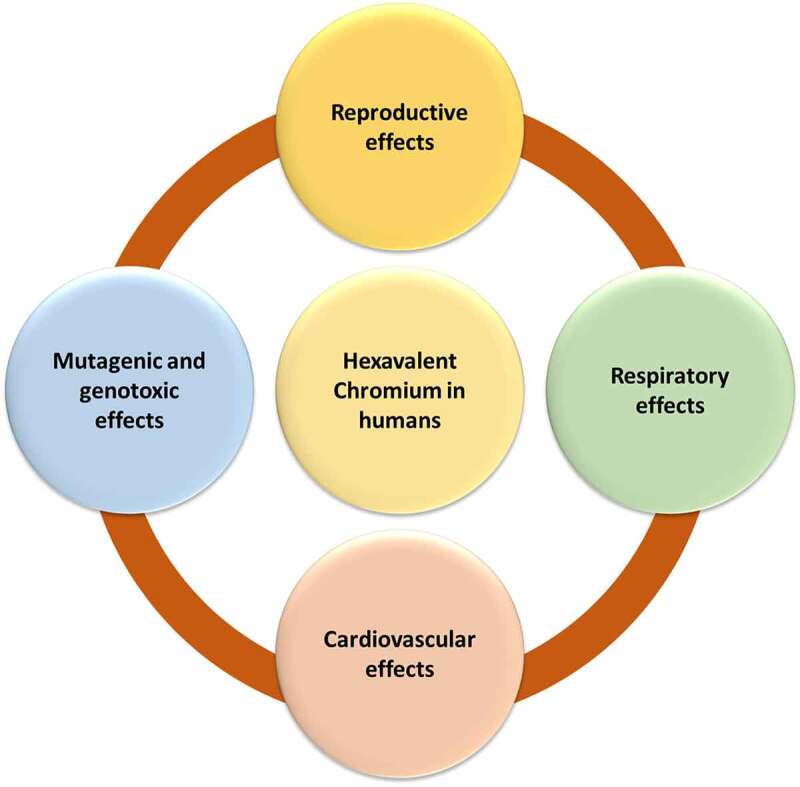
Toxicological effects of hexavalent chromium on humans.

**Figure 2.**
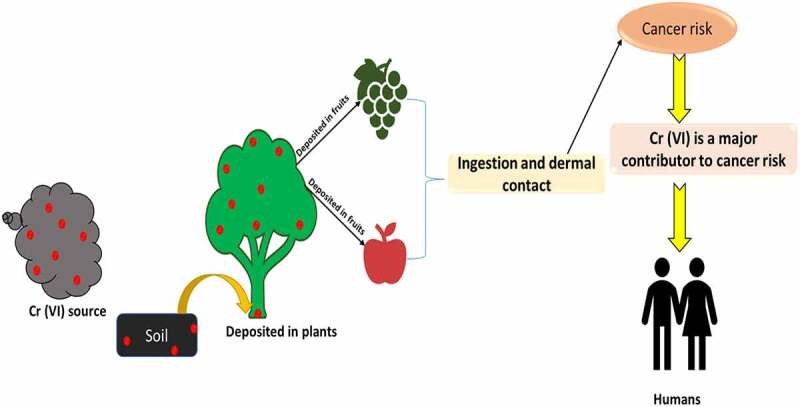
Hexavalent chromium (Cr (VI) has effects on the ecosystem and human health.

Severe and repeated exposure to chromium and related compounds, particularly those containing hexavalent ions, can cause health issues. The toxic effects of (Cr (VI)) on human health have been provided in Figure 1 & Figure 2.

### 2.1. Chromium (VI) effects on macrophages

Lung shape is unaffected by chromium inhalation, but macrophages become larger, multinucleated, or vacuolated, and nodules form in intra-alveolar spaces. Higher concentrations of Cr (VI) suppress alveolar macrophage phagocytic activity and the humoral immune response, whereas lower levels of Cr(VI) stimulate alveolar macrophage phagocytic activity and the humoral immunological reaction.

### 2.2. Chromium (VI) effects on immune response

An early study done on human cells by Borella et al., 1983 who studied the effects of Cr (VI) and other metals on cultured human lymphocytes found that chromium induces reductions in both blastogenesis and immunoglobulin production in relation to its capability to enter the cells.

### 2.3. Chromium (VI) induced cell death

Chromium has been shown to be cytotoxic to cells. Vasant et al., 2001 discovered that apoptosis is the method of cell death in human lymphocytes when Cr (VI) is present.

## 3. Hexavalent chromium effect on plant health

**Figure 3.**
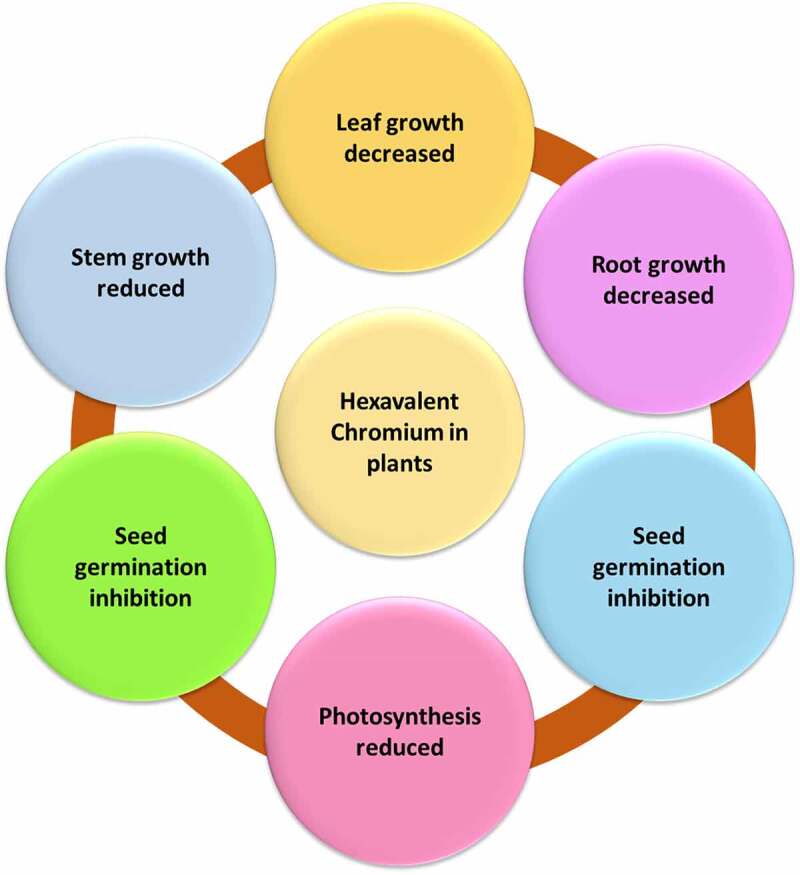
Toxicological effects of hexavalent chromium on the plant.

Plants show signs of Cr (VI) toxicity, including delayed seed germination, damaged roots reduced root growth, reduced biomass, reduced plant height, photosynthetic impairment, membrane damage, leaf chlorosis, necrosis, low grain production, and ultimate death of the plant. The most prevalent chromium compounds in soil are HCrO_4_ and CrO_42_, which are easily absorbed by plants and contaminate soil [41]. Because of its extremely low solubility, Cr (VI) has been found to cause significant injury to living tissue [42]. Plant shoot length and biomass are affected by Cr (VI) exposure. Though low levels of Cr (3.8104 M) do not affect some crops, chromium compounds are highly poisonous to most plants and damage their growth and production [43]. According to Elahi et al., 2020 [43], Cr (VI) can be severely harmful to plants at concentrations as low as 5 mg/kg in soils and 0.5 mg/L in solution. Cr (VI) is linked to a decrease in nutrient intake and photosynthesis, which contributes to the delayed growth of plants. Various physiological, structural, and biochemical processes in plant cells are severely disturbed, resulting in the generation of reactive oxygen species. Chlorosis and plant necrosis are two symptoms of Cr poisoning [44]. Chromium affects the growth of leaves, which are part of the photosynthesis organ of plants. Increasing chromium concentration causes a considerable decrease in leaf area and biomass, as well as decreased photosynthesis and the production of chlorophyll content and necrosis in leaves. Under Cr (VI) exposure, many destructive processes take place in leaves. Chlorophyll synthesis is suppressed, the chloroplast ultrastructure is disrupted, photosynthetic electron transport is inhibited, and magnesium ions are released from the chlorophyll molecule [45]. Reduced plant development, leaf deformation, and necrosis, root tissue damage, chlorosis, decreased enzyme activity, food uptake, transport, photosynthesis, lipid peroxidation, DNA strand break, and chromosome aberration are all symptoms of Cr (VI) toxicity in plants [46–48]. As a result, chromium (VI) can interfere with photosynthesis, seed germination, and nutrient intake, as well as the overall growth and functionality of the plant. The toxic effect of Cr (VI) on plants has been provided in Figure 3.

## 4. Hexavalent chromium (Cr (VI)) effect on microbiota

Chromium is the strongest metal in nature, ranking 17th in crust richness, and is primarily merged with the other elements to form trivalent and hexavalent compounds [49]. A variety of factors, including pathogens, habitat destruction, increased ultraviolet radiation, introduced non-native species, and contaminants, have all contributed to amphibian population declines. Intestinal microbial communities play a critical role in maintaining the host’s health [50]. They play a role in the regulation of numerous physiological functions [51]. Statistical analysis found that the human gut contains ~10^14^ bacteria, which is roughly 10 times the number of body cells [52].

## 5. Microbial remediation

Numerous microorganisms and plants have developed various strategies to counteract the toxic effects of Cr (VI) as shown in Table 1 [53–62]. Among the various methods, microbes’ enzymatic reduction of Cr (VI) into Cr (III) is the best-understood mechanism for such bioremediation [63]. For the detoxification of Cr (III) contamination and bioremediation of polluted waste, biological reduction of Cr (VI) to Cr is a potentially useful technique. Microbes that remove chromium from the environment offer a healthy and environmentally beneficial alternative to long-term manufacturing. These methods can be used to a wide range of microbial biomass, including bacteria, fungi, and algae. Biosorption of Cr (VI) has been proposed as a potential alternative to current industrial wastewater detoxification processes that use biomass (growing, resting, and dead cells), as well as biological and agricultural waste materials [64]. Chromium-resistant bacteria are responsible for or contribute to the biological reduction of Cr (VI) to less mobile Cr (III), and precipitation” of these bacteria could be a useful method for cleaning up polluted Cr (VI) areas. Through enzyme-catalyzed hazardous chemical degradation, bacteria successfully remove metal ions from the environment by using them as a source of energy and converting them to biomass [65]. Microbes in soil, underground materials, water, sludge, and residues are stimulated to break down environmentally detrimental substances to eco-friendly or acceptable levels during the microbial remediation process. The removal of chromium and other heavy metals from industrial effluents has been demonstrated using bioremediation strategies such as bioaccumulation, biotransformation, biosorption, and bioleaching [66]. Bioaccumulation is a metabolism-dependent method in which only live biomass uses cellular energy to transport hexavalent chromium (Cr (VI)) through the cell membrane. The bioaccumulation process in microorganisms is divided into the following phases. Initially, potentially hazardous heavy metal ions link themselves to the cell’s surface ligand. The metal-ligand combination that develops on the cell surface is subsequently transported inside by transporter protein. In addition, intracellularly transported complexes interact with metal-binding proteins such phytochelatins and metallothionein, causing precipitation, methylation, and other reactions. At the higher metal level, the approach only works on living cells and stops microbial cell development. Moreover, the biosorption, biotransformation, and bioaccumulation processes break down and remove harmful chromium ions from industrial effluent in an environmentally beneficial manner.

**Table 1.** Different physical, chemical, and biological methods available for chromium remediation [94]

S. No.	Biological Methods	Chemical Methods	Physical Methods
1.	Biosorption	Hydrogen sulfide (H_2_S)	Adsorption
2.	Bioaccumulation	Sodium metabisulfite (NaHSO_3_)	Membrane filtration
3.	Reverse osmosis	Ferrous sulfate (FeSO_4_)	Extracellular precipitation
4.	Bioreduction	Sodium dithionite (Na_2_ S2 O_4_)	Ion exchange
5.	Electrodialysis	Calcium polysulfide (CaS_5_)	Biomineralization

### 5.1. Biosorption of Cr (VI)

Biosorption, unlike bioaccumulation, is a metabolic rate-independent movement that can occur in both dead microbial biomass and living cells [67]. Potential hazardous ions, like Cr (VI), bind extracellularly to different functional groups of the microbial cell wall and are then eliminated by ion exchange, surface precipitation, or a complicated creation process [68]. The composition and design of bacteria’s cell walls differ. For example, bacterial cell walls are mostly peptidoglycan, fungi’s cell walls are mostly glucans, glycoproteins, chitin, melanin, and sulfonated polysaccharides, and algae’s cell walls are mostly alginate, sulfonated polysaccharides, and mannans [69]. The biosorption process’ mechanism is complicated since it is dependent on the types of biomass employed, the functional groups of the microbial cell wall, its structure, and the extracellular polymer compounds produced by bacteria [70,71]. For eliminating harmful heavy metals from polluted environments, the biosorption method is thought to have certain advantages over standard bioremediation strategies. The biosorption process has several advantages, including the presence of multifunctional groups and a homogeneous distribution of binding sites on the cell surface, biosorbent renewal, high efficiency, and the possibility of metal recovery. Because of these and other advantages, biosorption of heavy metals, notably hexavalent chromium, employing diverse microbial biomass has gotten a lot of attention. Environmental scientists, engineers, and biotechnologists have been fascinated by the ability of organisms to remove heavy metal ions induce their transformation to less dangerous forms for decades [72].

The Cr (VI) ion binds extracellularly to various functional groups of the microbial cell wall, which are eliminated via surface precipitation, ion exchange, or a similar mechanism. Microbes are organisms that have formed techniques for thousands of years to cope with environmental stress. Heavy metal-resistant defensive systems abound in microbial cells. The mechanisms involved are extracellular and intracellular sequestration, active metal ion transport, and metal ion reduction [73]. The biological process of heavy metal removal may be either biosorption or bioaccumulation, depending on the cell’s metabolism [74], the biological process of heavy metal elimination might be either biosorption or bioaccumulation. Increased membrane permeability increases intracellular uptake of heavy metals, which is a metabolism-dependent process. It can only happen in living creatures when pollutants are carried into the cell and metal ions accumulate inside the biosorbent’s cell [75]. Biosorption is a fast, autonomous, and metabolically passive mechanism that allows heavy metal ions to be selectively sequestered by dead/inactive biomaterials [76]. Heavy metals bind to cell walls extracellularly during biosorption, but once inside the cells, they bind to proteins such as metallothionein during the bioaccumulation process. A solid phase serves as the biosorbent in all of these biosorption processes. The sorbate is drawn and bonded by numerous mechanisms due to the sorbent’s increased affinity for the sorbate species [77].

**Figure 4.**
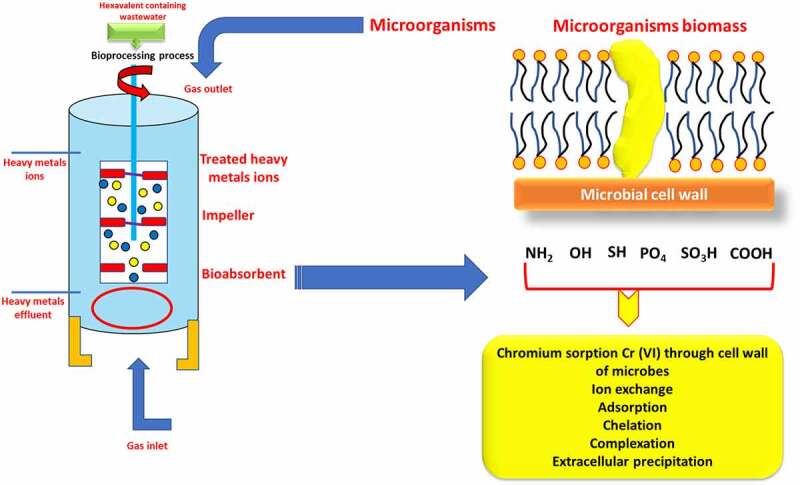
Hexavalent chromium (Cr (VI)) biosorption process by microorganisms.

Biosorption is a physiochemical contact between metal species and biological species’ cellular components. Accumulation, adsorption, oxidation, methylation, and decrease of poisonous, Cr (III) to Cr (VI) are some of the mechanisms underpinning their resistance. Heavy metal ions can become lodged in the cellular structure of these organisms and then absorbed via binding sites. Phosphates, carboxyl, imadizole, amino, hydroxyl moieties, thioether, sulfate, phenol, amine, and sulfhydryl groups are all found in biosorbents [77]. Microorganisms interact with metal ions in a variety of ways, including cell wall-associated metals, intracellular accumulation, metal siderophore, extracellular polymeric contacts with extracellular mobilization or immobilization of metal ions, transformation, and metal volatilization. Ion exchange, complexation, adsorption, and microprecipitation are all examples of physicochemical interactions between the charged surface groups of microbes and ions in solution [78]. In the case of bioaccumulation, metal sequestration or uptake is followed by metallothionein binding, metal (Cr) localization within cell components, extracellular precipitation, metal deposition, and complexation [79]. Chromium translocation into the cell, chromium binding to the cell surface, and Cr (VI) reduction to Cr are the three phases of microbial Cr (VI) removal (III). Microbes reduce Cr (VI) on the cell surface, outside the cell, or within the cell, either directly through chromate reductase enzymes or indirectly through Cr (VI) metabolite reduction [80]. Hexavalent chromium (Cr (VI)) biosorption process by microbes has been provided in Figure 4 & Table 2 here.

**Table 2.** Various microbes used for biosorption process of chromium (VI)

S. No.	Microorganisms	Remediation %	References
1.	*Chelatococcus daeguensis*	94.42 (%)	[82,95]
2.	*Pseudomonas alcaliphila* NEWG-2	96.60 (%)	[83,96]
3.	*Bacillus salmalaya*	20.35 mg/g	[84,97]
4.	*Bacillus sp*	75 (%)	[85,98]
5.	*Bacillus sp*	99 (%)	[86,99]
6.	*Klebsiella sp*	63.08 (%)	[87,100]
7.	*Sinorhizobium sp*. SAR1	285.71 mg/g	[88,101]
8.	*Aspergillus sp*	92 (%)	[89,102]
9.	*Aspergillus terreus*	54 (%)	[90,103]
10.	*Pleurotus ostreatus*	100 (%)	[91,104]
11.	*Pseudopediastrum boryanum*	70 (%)	[91,104]
12.	*Scenedesmus sp*	98 (%)	[92,105]
13.	*Chlorella colonials*	97.8 (%)	[93,106]
14.	*Spirulina platensis*	45.5 mg/g	[94,107]
15.	*Isochrysis galbana*	335.27 mg/g	[95,108]
16.	*Chlamydomonas sp*	91 (%)	[36,109]

**Table 3.** Various functional groups involved in chromium (VI) binding by different microorganisms

S. No.	Microorganisms	Functional groups	References
1.	*Bacillus marisflavi* and *Arthrobacter* sp	-OH, -NH acetamido group, free phosphates, phosphate groups, -CN	[34,110]
2.	*Pseudomonas aeruginosa* Rb-1 and *Ochrobactrum intermedium* Rb-2	-OH, -NH, S-, -C-C- and C-Cl,- carboxylic group,	[96,111]
3.	*Streptomyces werraensis* LD22	O–H or N–H, C–H, C–O –	[97,112]
4.	*Aspergillus foetidus*	C = O, C-Cl, PO_4 −3_ amine, N = C = S, OH, C-O	[98,113]
5.	*Pleurotus ostreatus*	NH and COOH	[99,114]
6.	*Aspergillus Niger*	-COOH, -OH, -NH_2_	[100,115]
7.	*Klebsiella sp*.	-NH_2_, O-H, -CONH-, -COOH, C = C, -CH_2_	[87,100]
8.	*Halomonas* sp. DK4	–OH, – CH_2_, N-H, P–O–C, C = O	[101,116]
9.	*Scenedesmus sp*	N-H, O-H, C-H, -COOH, C-F, C-Cl, C-Br, C-O	[31,117]
10.	*Chlorella miniata*	O-H and N-H, C-H, -CH_3_, COO-, P = O, C-O –	[102,118]
11.	*Arthrinium malaysianum*	–OH, C–O, C = O, – NO_2_, CxOH	[103,119]
12.	*Pleurotus ostreatus*	NH and COOH-	[99,114]
13.	*Aspergillus Niger*	-COOH, -OH, -NH_2_	[100,115]

**Table 4.** Efficiency and mechanism of different microbes for the removal of Cr (VI)

Microbes	Concentration (mg/L)	Carbon source	Temperature (°C)	pH	Efficiency	Mechanisms	References
*Serratia* sp. C8	20	Glucose	28	6–8	≈80%	Bioreduction	[120]
*Sporosarcina saromensis* M52	50–200	Acetate	35	7–8.5	>90%	Bioreduction	[121]
*Sphingopyxis macrogoltabida* SUK2c	4		31	2	55%	Biosorption	[122]
*Bacillus* sp. CRB-B1	100	Glucose	37	7	Completely	Bioreduction, biosorption	[123]
	100	Fructose	37	7	89.54%	Bioreduction, biosorption	[123]
	100	Sodium lactate	37	7	4.8%		[123]
*Pisolithus* sp1	25	Organic acid	30	5–6	99%	Bioreduction, biosorption	[124]
*Aspergillus* sp.	100	Sucrose	27	4	98.96%	Biosorption	[125]
*Leiotrametes flavida*	1000		30	6	72.38%	Biosorption	[126]
*Bacillus cereus*	200	Sucrose	37	7.5	Completely	Bioreduction, Biosorption	[127]

### 5.2. Hexavalent chromium to tetravalent chromium reduction

**Figure 5.**
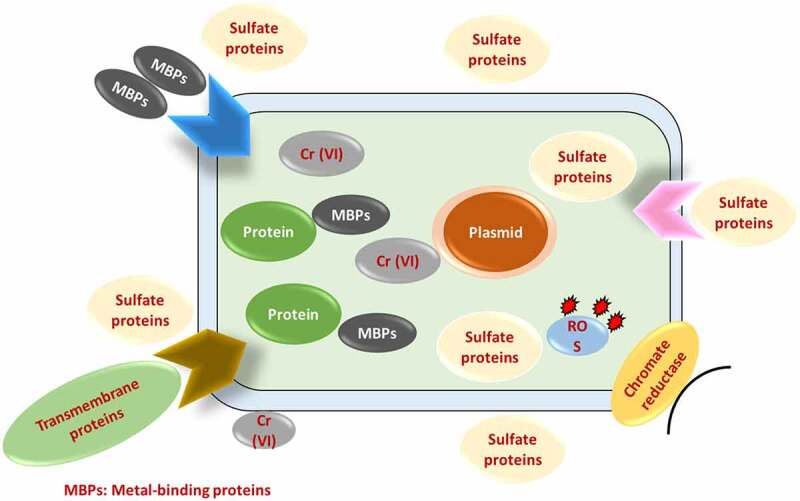
Cr(Ⅵ) detoxification mechanism.

On a local and big scale, textile, galvanizing, tannery, leather, metallurgical, electroplating, paint, and metal processing, and refining sectors produce dangerous metal ions in their effluents. By releasing metal ions into surrounding streams, rivers, and open pits, the above-mentioned businesses cause problems for the aquatic environment. Changes in surface and groundwater quantities are most likely to be experienced as a result of these metals’ potential impact on the environment [81]. Such hazardous elements not only endanger human health but also have an impact on other living things [82]. These source physical uneasiness and, intimidating illnesses, such as kidney damage and cancer [83]. Whereas its reduced trivalent form, (Cr^3+^) is less toxic, insoluble and a vital nutrient for humans. The high toxicity of Cr is stringent regulations are imposed on the release of Cr into surface water bodies to below 0.05 mg/l by the U.S. EPA and the European Union, while total Cr forms to below 2 mg/l [84]. Cr (Ⅵ) detoxification mechanism has been provided in Figure 5 & Tables 3, 4.

## 6. Factor affecting bioremediation

The process of bacteria, fungi, and plants digesting, altering, immobilizing, and removing countless hazardous contaminants from the environment is known as biological treatment. Microbes are engaged because their enzymatic pathways operate as biocatalysts, allowing biochemical reactions to proceed more quickly and destroy the targeted contaminant. Microbes have access to a diversity of materials substances to assist them to manufacture nutrients and energy to build more cells so they act against pollution. The chemical composition and concentration of contaminants, as well as the physico-chemical features of the environment and their availability to microbes, all influence bioremediation efficacy [85]. Because bacteria and contaminants do not come into contact with each other, the rate of deterioration is influenced. Furthermore, bacteria and contaminants are not evenly distributed across the environment. Due to a variety of elements, regulating and improving bioremediation procedures is a complicated system. The availability of contaminants to the microbial community, and the presence of a bacterial community capable of decomposing the toxic pollutants.

### 6.1. Availability of nutrients

Nutrient supply influences the critical nutritional balance for microbial growth and reproduction, as well as the rate and effectiveness of biodegradation. By adjusting the bacterial C: N: P ratio, particularly the delivery of important nutrients like P and N, can boost degradation competence. Microorganisms require a variety of nutrients, including carbon, nitrogen, and phosphorus, to endure and remain their activity. The degree of hydrocarbon breakdown is similarly limited at low concentrations. In cold conditions, adding a suitable amount of nutrients is a good technique for enhancing microorganism metabolic activity and hence biodegradation rate. The availability of nutrients limits biodegradation in aquatic environments [86]. Such nutrients can be found in the natural world, although in little amounts [87].

### 6.2. Environmental factors

All through the procedure, the metabolic capabilities of the microorganisms and the physico-chemical features of the targeted pollutants determine probable interactions. The ambient variables at the interaction location, on the other hand, impact the actual success of the interaction between the two. Temperature, site features, solubility in water, redox potential, nutrients, pH, moisture, soil structure, and oxygen content, as well as physico-chemical bioavailability of contaminants and all influence microorganism development and activities. The parameters listed above determine the kinetics of deterioration [88,89]. Bioremediation can occur in a wide pH range; however, in most aquatic and terrestrial systems, a pH of 6.5 to 8.5 is generally ideal for microbial degradation. Pollutant metabolism is influenced by the types and amounts of soluble materials present, as well as the pH of terrestrial and aquatic ecosystems [90].

Temperature is the most essential physical factor in determining microbe survival and hydrocarbon content [91]. The sub-zero temperature of the water in this region causes transport channels within microbial cells to shut down or even freeze, rendering them metabolically dormant, rendering most oleophilic bacteria metabolically inert [92,93]. The metabolic cycle of biological enzymes involved in the degradation process has an optimal temperature and will not be the same at all temperatures. Furthermore, the degradation of a certain substance necessitates a specific temperature. Temperature influences microbial physiological features; hence it can speed up or slow down the bioremediation process. The rate of microbial activity increases as the temperature rises, peaking at the optimal temperature. It began to diminish abruptly when the temperature increased or decreased, eventually coming to a halt after reaching a set degree. The pH of a chemical, which refers to its acidity, basicity, and alkalinity, has an impact on microbial metabolic activity and the rate at which it is eliminated. The potential for microbial growth in the soil can be determined by measuring pH. Increase or decrease pH values resulted in poor results; metabolic processes are extremely sensitive to even minor pH variations. Because hazardous characteristics of some contaminants are present in high concentrations, toxic effects on microorganisms might occur, slowing clean-up. The degree and processes of toxicity differ depending on the toxicants, their concentrations, and the microbes exposed. Targeted life types are poisonous to some organic and inorganic chemicals [88].

## 7. Future prospects

The challenging problem of removing Cr (VI) pollutants from the environment has resulted in the development of numerous bioremediation strategies, particularly competent reduction techniques by microbes. Microbial degradation is a very profitable and appealing technology for cleaning, controlling, and restoring contaminated habitats via bacterial metabolism. Through autogenous enzymes or externally added reducing substances, microorganisms provide electrons to reduce Cr (VI). The rate at which undesired waste chemicals degrade is determined by competition with biological agents, insufficient food supply, unpleasant external abiotic conditions (aeration, moisture, pH, temperature), and limited pollutant bioavailability. Because of these characteristics, biodegradation under natural conditions is less successful, resulting in less favorable effects. Because bioremediation is only successful when the environment allows for microbial growth development. Bioremediation has been employed in a variety of locations around the world with variable degrees of effectiveness. In most instances, the benefits outweigh the disadvantages, as seen by the expanding number of sites that use this technology and its growing popularity over time. Usually, many species from various areas are researched and determined to be effective regulatory processes. Previous reports on bioremediation advanced technologies focused primarily on water bodies; however, arable land is currently suffering from severe heavy metal pollution, so future microbial remediation technology should also target the soil and environment. In response to the research deficiencies proposed, the following recommendations are made:

1. This is difficult to achieve the goal of governance using purely cultured microbes due to the complexity of the actual environmental conditions, particularly the soil. Through the synergy of microbes, the use of mixed cultures of microorganisms can improve the ability to adapt to the environment and treatment effectiveness.
2. Because polluted sites contain more than one type of heavy metals, this is appropriate to screen microbes for their potential to reduce or bind numerous toxic metals.
3. Bioremediation has a lower performance than physical and chemical materials, and it also takes a long time to remove heavy metals. Future studies could concentrate on the combination of microbes in the formation of a consortium to improve process efficiency.

## 8. Conclusions

The bioremediation and biosorption of chromium (VI) by microorganisms are discussed in this paper, as well as the parameters that metal accumulation mechanisms impact on metal’s elimination. The microbial remedy of Cr (VI) is among the most efficient and protracted strategies for decreasing excess Cr (VI) levels in the ecosystem. To survive in such a hazardous environment, these microorganisms have evolved amazing systems to maintain equilibrium and resistance to toxic metals. The biosorption technique is a microbe technology for eliminating chromium from the aquatic environment, it is safe and cost-effective, and has a great deal of potential in terms of future applications. The process of biosorption requires transport across the precipitation, complexation, cell membrane, ion exchange, and physical adsorption. The pH, contact time, temperature, biomass, and metal concentration parameters can all affect the biosorption ability of the biosorbent. Because industrial wastewaters, unlike laboratory solutions, hazardous heavy metals, simultaneous removal of multiple coexisting contaminants may be difficult. Conclusion of this review more research on this topic is required to fully exploit the benefits of microbial biotechnology in the ecosystem.
